# Atomic mechanism of polarization-controlled surface reconstruction in ferroelectric thin films

**DOI:** 10.1038/ncomms11318

**Published:** 2016-04-19

**Authors:** Peng Gao, Heng-Jui Liu, Yen-Lin Huang, Ying-Hao Chu, Ryo Ishikawa, Bin Feng, Ying Jiang, Naoya Shibata, En-Ge Wang, Yuichi Ikuhara

**Affiliations:** 1 Electron Microscopy Laboratory, School of Physics, Center for Nanochemistry, Peking University, Beijing 100871, China; 2 Collaborative Innovation Center of Quantum Matter, Beijing 100871, China; 3 Department of Materials Science and Engineering, National Chiao Tung University, Hsinchu, Taiwan 30010, China; 4 Institute of Physics, Academia Sinica, Taipei, Taiwan 105, China; 5 Institute of Engineering Innovation, The University of Tokyo, Tokyo 113–8656, Japan; 6 International Center for Quantum Materials, School of Physics, Peking University, Beijing 100871, China; 7 Nanostructures Research Laboratory, Japan Fine Ceramic Center, Nagoya 456-8587, Japan; 8 WPI Advanced Institute for Materials Research, Tohoku University, Sendai 980-8577, Japan

## Abstract

At the ferroelectric surface, the broken translational symmetry induced bound charge should significantly alter the local atomic configurations. Experimentally revealing the atomic structure of ferroelectric surface, however, is very challenging due to the strong spatial variety between nano-sized domains, and strong interactions between the polarization and other structural parameters. Here, we study surface structures of Pb(Zr_0.2_Ti_0.8_)O_3_ thin film by using the annular bright-field imaging. We find that six atomic layers with suppressed polarization and a charged 180° domain wall are at negatively poled surfaces, no reconstruction exists at positively poled surfaces, and seven atomic layers with suppressed polarization and a charged 90° domain wall exist at nominally neutral surfaces in ferroelastic domains. Our results provide critical insights into engineering ferroelectric thin films, fine grain ceramics and surface chemistry devices. The state-of-the-art methodology demonstrated here can greatly advance our understanding of surface science for oxides.

## Abstract (web summary)

Miniature of electronic devices is attractive yet challenging due to structural variation at nanoscale. Here, Gao *et al*. report atomic imaging of reconstruction and unusual domain walls on Pb(Zr_0.2_Ti_0.8_)O_3_ surfaces, providing possibilities to engineer nanoscale structural change.

## Graphical abstract

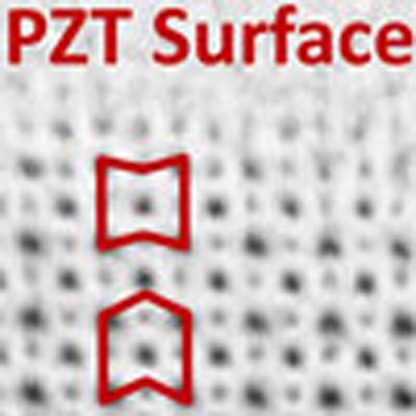

At a surface of ferroelectrics^1^,^2^,^3^, the abrupt change in the coordination number significantly affects the structural parameters such as the lattice, strain and charge, thus altering the neighbouring polarization/ferroelectricity^4^,^5^,^6^,^7^,^8^,^9^ that is generated by the delicate balance between long-range Coulomb and short-range covalent interactions^10^,^11^. For example, the subsurface polarization-induced bound charge must cause re-distribution of free carriers and/or structural distortion^11^ at surfaces to minimize the total energy, forming surface reconstructions. The presence of surface skin layers with different nature than the bulk^4^,^12^,^13^,^14^ (for example, surface dielectric dead layer) can significantly influence the properties of ferroelectric devices or even dominate the entire response. Thus, revealing the surface structure and relation with the subsurface is vital for applications in electronic devices and lies at the heart of the ferroelectric surface chemistry^12^,^15^,^16^,^17^.

Despite a lot of simulation efforts have so far been devoted and predicted various properties of ferroelectric surfaces^4^,^5^,^6^,^7^,^8^, only few experimental studies can be found^9^,^18^. By using those bulk-based techniques such as the X-ray characterization^9^,^18^, it is extremely difficult to extract the information of surface structures or determine the relation with subsurface structures because of the strong spatial variety between nano-sized domains and ultrathin thickness of surface reconstruction layers. On the other hand, the commonly used surface probe technique scanning tunnelling microscopy (STM) is usually not suitable to study these insulator-like ferroelectric oxides and unable to obtain the information underneath the surface. The details of a ferroelectric surface layer (including both the topmost surface skin and nearby subsurface structure) such as the polarization, strain and octahedral tilt, therefore, remain exclusive. Recently, the advancements of the annular bright-field (ABF) imaging in an aberration corrected scanning transmission electron microscope^19^,^20^,^21^ have made it possible to simultaneously determine both the heavier cation and lighter oxygen positions in oxides such as SrTiO_3_ (refs 19, 20), Fe_3_O_4_ (ref. 19), TiO_2_ (ref. 20) and Al_2_O_3_ (ref. 21), allowing us to precisely measure the structural parameters at both the surface^22^ and subsurface of functional materials irrespective to their conductivities.

Here, we study surface structures in different domains in Pb(Zr_0.2_Ti_0.8_)O_3_ (PZT, ref. 23) thin film to uncover the structural parameter changes occurring on the surface layer associated with the polarization underneath the surface. We find that the surface structure is governed by the polarization underneath. A ferroelectric dead layer with suppressed polarization and expanded lattice ∼4.7%, and a charged 180° domain wall exist on the negatively poled surface with six atomic layers thick (equivalent to three unit cells). The positively poled surface remains the same with the bulk-like subsurface region. The ferroelastic domain with the polarization parallel to the surface has a seven-atomic-layer-thick reconstruction layer in which the lattice constant is contracted ∼2.5% and the in-plane polarization gradually decreases and rotates towards to the free surface, forming an unusual charged 90° domain wall in the (100) plane. Such a charged 90° domain wall has never been observed before as the regular 90° domain wall should be in the (110) plane. The formation of complicated surface polarization configurations in different domains is driven by the screen mechanism of the bound charge at the surfaces. The presence of ferroelectric dead layer on surfaces can explain the failure phenomena in ultra-thin ferroelectric devices observed in the past experiments. The subsurface polarization controlled surface reconstructions and properties may also allow us to engineer ferroelectric surfaces for some novel electronic devices and surface catalysis. The ability to simultaneously determine the structures of surface and subsurface provides a unique and powerful method to explore the surfaces of functional materials in particular for those insulators, which are usually not suitable for STM study.

## Results

### Negatively poled surface

**Figure 1:**
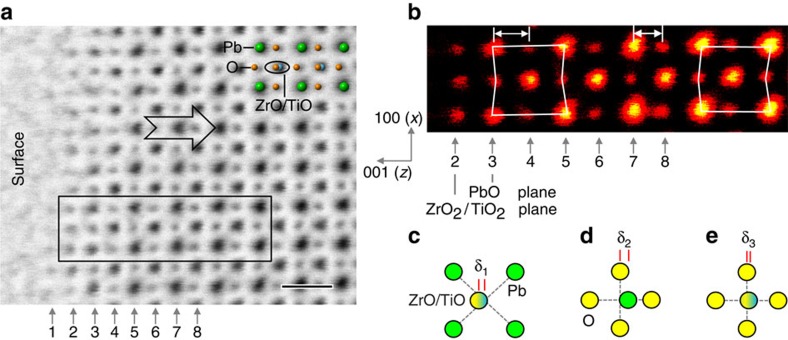
Atomic structure of surface in Pb(Zr_0.2_Ti_0.8_)O_3_ thin film. (**a**) An atomically resolved annular bright-field (ABF) image of the negatively poled surface. The arrow indicates the polarization is downward in this domain. The viewing direction is along [010]. The atomic layers are labelled with the numbers. A schematic is overlaid with the ABF image in the up-right corner. Scale bar, 0.5 nm. (**b**) The enlarged view of the rectangle region in **a**. The contrast is inverted for clarity. The white outlines show that the oxygen at the 3rd layer shifts to the right direction opposite to the oxygen shift at the 5th layer. (**c**) Schematic showing that δ_1_ is the relative displacement of ZrO/TiO column respective to the four neighbouring Pb columns. (**d**) Schematic showing that δ_2_ is the relative displacement of Pb column respective to the four neighbouring O columns. (**e**) Schematic showing that δ_3_ is the relative displacement of ZrO/TiO column respective to the four neighbouring O columns.

Figure 1a shows an ABF image of a negatively poled surface in a PZT thin film with the viewing direction of [010], from which both the cation (Pb and ZrO/TiO) and oxygen columns are visible and the topmost layer is PbO. In PZT, the ferroelectricity originates from the strong covalent Pb–O bond, that is, the shorter Pb–O bond corresponds to the larger polarization^24^. Thus, the polarization in this region is downward (pointing towards right) as the O shift to the left Pb column in Fig. 1b. The surface structure within the topmost two unit cells is significantly distinct from the bulk-like region: the O in the 3rd atomic layer shift to the right Pb plane, which is opposite to that in the bulk-like region such as 5th, 7th and 9th layers. Along the *c* direction (*z* direction), the opposite O displacements between 3rd and 5th layers cancel each other, leading to the reduced/zero polarization within this unit cell, which is well known as a ferroelectric dead layer. Such a dead layer has also been observed at the interfaces^25^, grain boundary^26^ and charged domain walls^27^. To reveal the detailed changes in the structural parameters near the surface, we calculate various relative distances between the interatomic columns, including the relative displacement of ZrO/TiO column respective to the four neighbouring Pb columns in Fig. 1c, relative displacement of Pb column respective to the four neighboruing O columns in Fig. 1d and relative displacement of ZrO/TiO column respective to the four neighbouring O columns in Fig. 1e.

**Figure 2:**
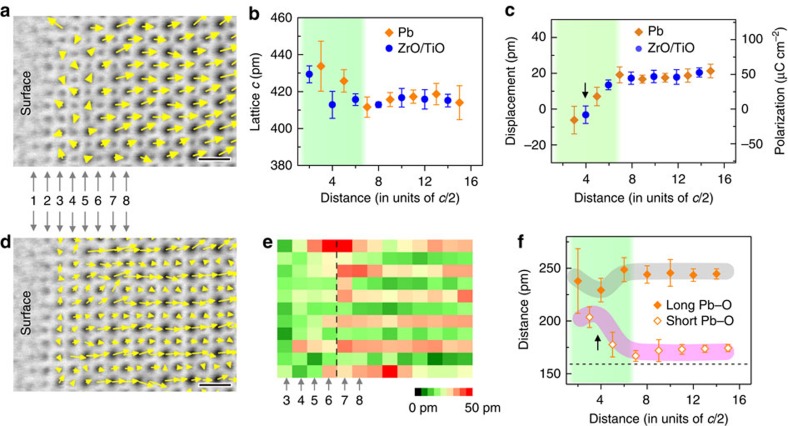
Structure of the negatively poled surface. (**a**) Displacement vectors between the Pb and ZrO/TiO columns overlaid with the ABF image. The yellow arrows represent displacement vectors. The atomic layers are labelled by the numbers. Scale bar, 0.5 nm. (**b**) Mean of the lattice parameter in [001] direction. Six data points were averaged to calculate the Pb lattice and five data points were averaged to plot ZrO/TiO lattice. The error bar is the s.d. The green colour highlights the surface region with different atomic configuration. (**c**) The *z*-component of displacement and polarization. Six data points were averaged to plot Pb displacement and five data points were averaged to plot ZrO/TiO displacement. The error bar is the s.d. (**d**) Vector map of the displacement between the cation and anion columns. Scale bar, 0.5 nm. (**e**) The magnitude map of the displacement between the cation and anion columns. The black dashed line highlights distinct magnitude between the left surface zone and right bulk-like region. (**f**) The long and short bond length of Pb–O along *z*-direction. Six data points were averaged to plot the Pb–O bond length. The error bar is the s.d. The pink band highlights the shorter Pb–O length, which directly represents the magnitude of polarization in PZT. The grey band highlights the longer Pb–O length. The dashed line 159 pm is calculated from the structure model in ref. 23. The arrow indicates the position of the charged 180° domain wall.

Figure 2a is the calculated vector map of the displacement between Pb and ZrO/TiO columns^28^ (see also Supplementary Fig. 1 for details), which represents the polarization distribution in the perovskite ferroelectrics^29^. From 3rd to 6th layer, the polar vectors have smaller magnitude and more random orientation. The lattice parameter *c* expands in the Fig. 2b, leading to an increase of tetragonality in the surface layer. The plot of the average *z*-component of displacement in Fig. 2c shows that zero net displacement exists at the 4th TiO/ZrO layer, whereas the negative displacement occurs in the 3rd layer, indicating the 4th layer acts as a charged 180° domain wall separating the tail-to-tail polarization configurations. Approximately, the polarization can be deduced from the displacements^28^,^29^,^30^: *P*∝Δ*z*, where *P* is the spontaneous polarization and Δ*z* is the displacement. Thus, the polarization is calculated to be^28^,^29^,^30^ ∼50 μC cm^−2^ in the bulk and linearly decreased to zero at 4th layer, and then increased to ∼20 μC cm^−2^ with the opposite direction.

To extract the other structural parameters such as oxygen octahedral distortion at the surface, the relative displacement vectors between the cations (Pb and ZrO/TiO) and anion columns (indicated in Fig. 1c) is calculated in Fig. 2d and the corresponding magnitude map is plotted in Fig. 2e. Similarly, the displacement vectors at the surface are smaller in magnitude and more random in orientation (see also Supplementary Fig. 2 for details). The plot of Pb–O bond length in Fig. 2f, which directly corresponds to the strength of ferroelectricity^24^, further confirms smaller net polarization at the surface and a charged 180° domain wall exists at the 4th layer. Such surface reconstruction is representative for the negatively poled surface, of which we have also confirmed in other locations in the same sample (see Supplementary Fig. 3 for details).

### Positively poled surface

**Figure 3:**
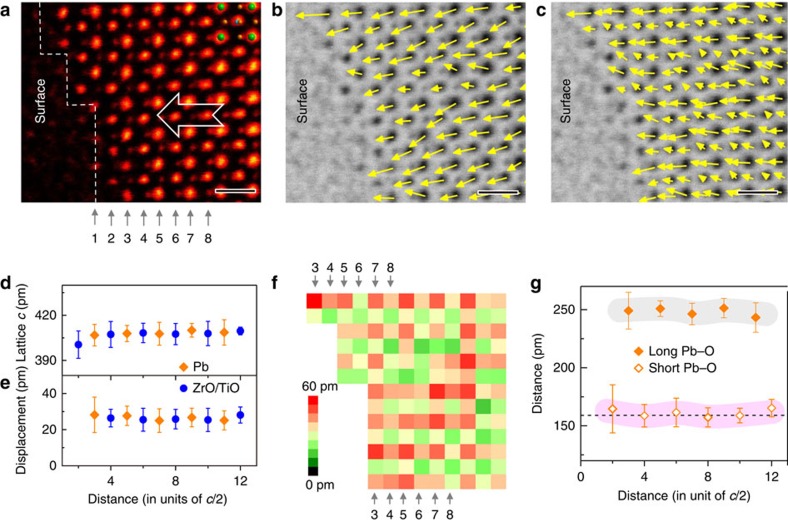
Structure of the positively poled surface. (**a**) An ABF image of the positively poled surface. The contrast is inverted and coloured for clarity. The arrow indicates the polarization is upward in this domain. Scale bar, 0.5 nm. (**b**) The vector map of the displacement between the Pb and ZrO/TiO columns overlaid with the ABF image. Scale bar, 0.5 nm. (**c**) The vector map of the displacement between the cation and anion columns. Scale bar, 0.5 nm. (**d**) Mean of the lattice parameter *c* near the surface. Seven data points were averaged to plot Pb lattice and six data points were averaged to plot ZrO/TiO lattice. The error bar is the s.d. (**e**) Mean of the *z*-component displacement between the Pb and ZrO/TiO columns. Seven data points were averaged to plot Pb displacement and six data points were averaged to plot ZrO/TiO displacement. The error bar is the s.d. (**f**) The magnitude map of the displacement between the cation and anion columns. (**g**) The long and short bond length of Pb–O along the *z*-direction. Seven data points were averaged to plot Pb–O bond length. The error bar is the s.d. The pink band highlights the shorter Pb–O length, which directly represents the magnitude of polarization in PZT. The grey band highlights the longer Pb–O length. No distinguishable difference is observed between the surface and subsurface. The dashed line 159 pm is calculated from the structure model in ref. 23.

Figure 3a is an ABF image of a positively poled surface in a domain with the downward polarization. In contrast, there is no distinguishable difference between the surface and bulk-like subsurface based on both polar vector maps in Fig. 3b,c (see also Supplementary Figs 4 and 5 for details). Constant lattice parameter, displacements and Pb–O bond length in Fig. 3d–g suggest no dielectric dead layer at the surface. Positively poled surface taken from another location of the same sample in Supplementary Fig. 6 shows the same phenomenon.

### Ferroelastic domain surface

**Figure 4:**
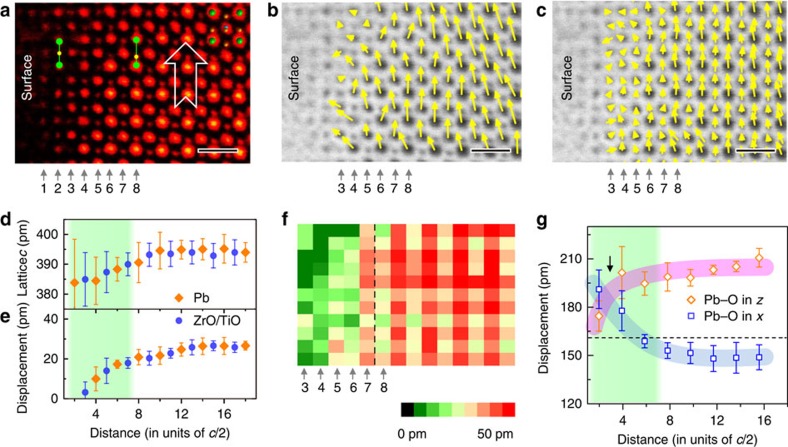
Surface structure of a ferroelastic domain with the in-plane polarization. (**a**) An ABF image of the ferroelastic domain. The contrast is inverted and coloured for clarity. The arrow indicates the polarization is parallel to the surface in this domain. Scale bar, 0.5 nm. (**b**) The vector map of the displacement between the Pb and ZrO/TiO columns overlaid with the ABF image. Scale bar, 0.5 nm. (**c**) The vector map of the displacement between cation and anion columns. Scale bar, 0.5 nm. (**d**) Plot of the lattice parameter *c* shows contraction on the surface. Six data points were averaged to plot Pb displacement and five data points were averaged to plot ZrO/TiO displacement. The error bar is the s.d. (**e**) Plot of the displacement between the Pb and ZrO/TiO columns. Six data points were averaged to plot Pb displacement and five data points were averaged to plot ZrO/TiO displacement. The error bar is the s.d. (**f**) The map of displacement between the cation and anion columns. (**g**) The short bond length of the Pb–O along *x*-direction and *z*-direction. Six data points were averaged to plot Pb–O bond length. The error bar is the s.d. The pink band highlights the shorter Pb–O length of the *z*-direction, and blue band highlights the shorter Pb–O length of the *x*-direction. The dashed line 159 pm is calculated from the structure model in ref. 23.

In addition, ferroelastic (90°) domains with the in-plane polarization (along [100] direction), whose surface is nominally neutral, can also be observed (see also Supplementary Fig. 7). The ABF image in Fig. 4a shows that the reconstruction still exists on such a surface: more uniform Pb–O bond length on the surface suggests a reduction in the in-plane polarization (*x*-component). The polar vectors in Fig. 4b,c (see also Supplementary Figs 8 and 9) show smaller magnitude of polar vectors above the 7th layer. Moreover, the vectors in the 3rd (TiO_2_/ZrO_2_) and 4th (PbO) layers appear disorder and have an out-of-plane component (*z*-component). The measured lattice parameter along the [001] direction (in Fig. 4d), however, exhibit a contraction at the surface: the interatomic distance in the cation sublattice decreases from 395 pm at the 8th layer to 385 pm at the 3rd layer (equivalently to ∼2.5% contraction). The plots of the displacements in Fig. 4e,f confirm that the thickness of surface zone is about seven atomic layers. Although the in-plane polarization starts to decrease from the 7th layer in Fig. 4g, the out-of-plane polarization appears only after the 4th layer, indicating a charged 90° domain wall exists between 3rd and 4th layer.

## Discussion

These distinct surface structures in different domains can be explained by the subsurface polarization-induced bound charge at the surface, which requires to be screened by either structural distortion, electronic reconstruction or re-distribution of free carriers. On the negatively poled surface, the holes must move to the *p* states of surface oxygen, or alternatively, oxygen vacancies should form on the surface acting as positively charged defects that contribute to screen the depolarization field. For the perovskite oxide, on the surface the formation energy of oxygen vacancy is usually lower because of the asymmetric bonding. With high-density oxygen vacancies at the surface, the bound charge can be effectively screened but meanwhile the structure prefers to expand the lattice because of the increased Coulomb repulsion between cations^31^. The measured reconstruction thickness of six atomic layers is basically in agreement with previous calculations^8^ of PbTiO_3_ for which four atomic layers reconstruction may exist on the negatively poled surface. However, the experimental observations indicate the surface reconstruction is even thicker and much more complicated containing charged domain walls.

The scenario for positively poled surface is different. The first principles calculation of PbTiO_3_ proposed only minor reconstruction about one atomic layers is required on the positively poled surface if there is extra adsorbed oxygen or Pb vacancies on the topmost surface to form a non-stoichiometric surface^8^. Since practically the Pb is volatile^9^ and the ABF image was recorded in a vacuum chamber (2 × 10^−5^ Pa), it is likely that the Pb vacancies act as negatively charged defects to compensate the positively bound charge.

For a perfect ferroelastic domain with polar vectors parallel to the surface, the surface is neither positively nor negatively charged and therefore no surface reconstruction is expected^7^. However, simulation suggested that near an atomic step on the surface, the in-plane polarization (along [100]) would be suppressed and meanwhile the out-of-plane polarization would be generated^7^ because the covalent Pb–O bonds changes and rotates the polar vectors. Such structural distortion can propagate as deep as three unit cells into the interior^7^, which is very close to our experimental observations of seven atomic layers reconstruction. Since at the surface, the formation of the oxygen and Pb vacancies^9^ is facile, the topmost unit cell is always rich in cationic and/or anionic vacancies with many tiny atomic steps. These steps influence the configuration of the neighbouring Pb–O bonds near the surface, generating an unusual charged 90° domain wall in (100) plane in Fig. 4, although the regular 90° domain wall in tetragonal ferroelectrics should occur in the (110) plane instead of (100) (ref. 32). However, it should be noted that on the positively or negatively poled surface, at such atomic steps the polar vector rotation is not favourable because these atomic steps do not break the polar vector continuity.

The thicknesses of the surface reconstruction ranging from zero to seven atomic layers is much smaller than that predicted from the mesoscopic measurements^18^. The polarization prefers to point towards the free surface irrespective to the electric dipoles at the subsurface, suggesting the non-stoichometric surface of our sample can locally stabilize the upward polar vectors. The *c*-lattice parameter at the negatively poled surface expands, whereas in the ferroelastic domain it contracts. In both of the cases, the tetragonality increases while ferroelectricity decreases. Overall, the surface structure is very distinct from the bulk-like subsurface and largely governed by the subsurface polarization via strong interactions between the structural parameters. Furthermore, the conclusion of polarization-dependent surface reconstruction is unlikely affected by the thin-surface amorphous layer or tiny specimen alignment (more details in the Supplementary Fig. 10 and Supplementary Discussion).

In summary, our results unambiguously show that the surface structure is sensitive to the polarization underneath. The reconstructed surface layers should have different physical and chemical properties, such as Fermi level^8^ and phase diagram^18^, thereby is expected to significantly influence the response of ferroelectric devices particularly in the ultra-thin film^33^ or very fine grain ceramics^34^. For example, the presence of the ferroelectric dead layer on the surface suggests that for the practical devices there is a critical thickness, below which the device does not function^35^. These findings also provide valuable insights into engineering of ferroelectric surface for the surface chemistry applications^8^,^12^. Although our observations are only based on the Pb(Zr_0.2_Ti_0.8_)O_3_, similar polarization-controlled surface reconstruction also likely applies to the other ferroelectrics due to the similarities in the bound charge screening mechanism. Furthermore, previous knowledge on the ferroelectric surface is mainly from the theoretical calculation. The experimental observations in our work indicate the surface reconstruction is much more complicated than the theoretical predictions, for example, unusual domain walls can exist in the ultra-thin surface reconstruction layers. This experimental methodology demonstrated in this work enabling picometre-scale measurement of structural parameters simultaneously for both of the surface and subsurface irrespective to the conductivity of materials can greatly advance our understanding of the surface structures and properties in the functional materials particularly for those insulators, which may be not suitable for the STM study.

## Methods

### Materials and characterization

Tetragonal PZT films oriented in the [001] direction were grown on (001) SrTiO_3_ substrate by pulsed laser deposition. No post treatment was performed to the thin films. The thickness of as-grown PZT film is ∼28 nm. The upward, downward and ferroelastic domains can be found in the cross-sectional transmission electron microscope specimens. The surfaces are typically more than 10 nm (25 unit cells) far away from the substrate in the present scanning transmission electron microscopy images to rule out the substrate effects. Cross-sectional transmission electron microscope specimens were thinned less than 30 μm by mechanical polishing and followed by argon ion milling in a Precision Ion Polishing System 691 (Gatan). Ion milling procedure consists of two steps. In the first stage of coarse milling, the guns were at 4 keV with angles 5° and −5°. In the following condition, the guns were set at 1 keV for 5 min with angles of 3.5° and −3.5°, and further lowered to 0.1 keV for 2 min for final surface cleaning. ABF images were recorded at 300 kV in JEM ARM300CF (JEOL Ltd.). The convergence semi-angle for imaging is 24 mrad, collection semi-angles snap is 12–24 mrad for ABF imaging. All high-resolution ABF images used for distance calculation in this work were raw data without any post filtering.

### Scanning transmission electron microscopy image analysis

Atom positions were determined by simultaneously fitting with two-dimensional Gaussian peaks to an *a priori* perovskite unit cell using a MatLab code^29^. In our definition, all the atom columns in the second layer (#2) should be visible and thus can be fitted with Gaussian peaks for distance calculation. In other words, in the first atomic layer (#1), there are at least part of atomic columns are not distinguishable in the ABF image. The lattice constant and bond-length for atomic columns in the #*n* layer are the distance between the #*n* and #(*n+1*) layer, where *n*≥2. Displacement vectors of the A column were measured relative to the centre of the surrounding B columns in the ABF images, where A and B can be Pb, ZrO/TiO and O columns. The relative displacements of atomic columns in the #*n* layer are calculated from the position values of #(*n−1*), #*n* and #(*n+1*) layers, where *n*≥3. Multislice simulation of ABF images was carried out by using a commercial software from HREM Research, Inc. The inner and outer angles were set to 12–24 mrad for the ABF detector and the aperture was set to 24 mrad. The electron beam directions was set as [0, 1, *α*], where *α* is the misalignment angle. Different thicknesses were tested and compared with experimental images to find the most suitable thickness value and 20 nm was used to calculate the misalignment effects.

## Additional information

**How to cite this article:** Gao, P. *et al*. Atomic mechanism of polarization-controlled surface reconstruction in ferroelectric thin films. *Nat. Commun.* 7:11318 doi: 10.1038/ncomms11318 (2016).

## Supplementary Material

Supplementary Information Supplementary Figures 1-10, Supplementary Discussion and Supplementary References
