# Supplementary material for: Atomic mechanism of polarization-controlled surface reconstruction in ferroelectric thin films
Source: Nat Commun. 2016 Apr 19;7:11318. doi: 10.1038/ncomms11318 (PMC4838897; doi:10.1038/ncomms11318)
Supplement: Supplementary Information — Supplementary Figures 1-10, Supplementary Discussion and Supplementary References [file ncomms11318-s1.pdf]

Supplementary Figures.

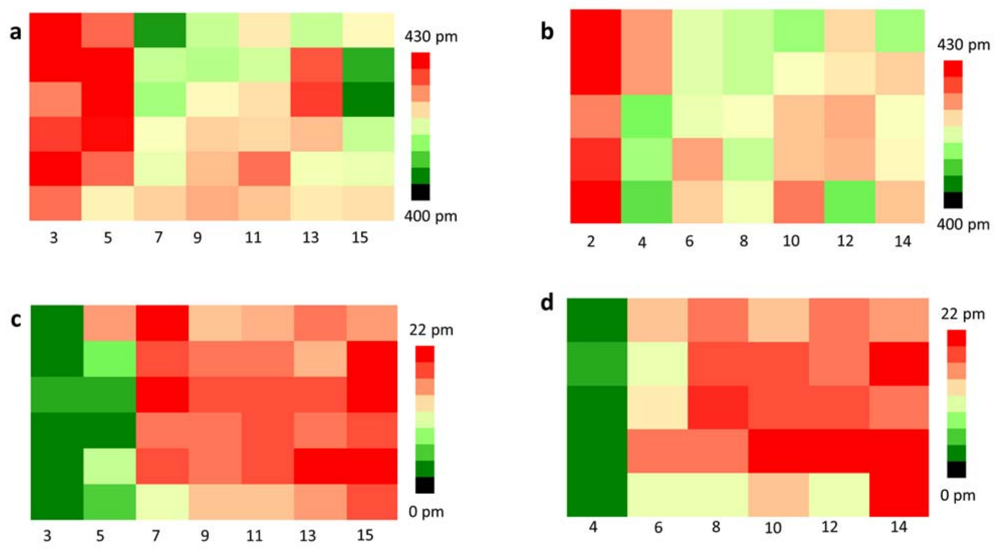

Supplementary Figure 1. | Structure of the negatively poled surface calculated from the cation sublattice. (a) The lattice parameter *c* map calculated from the Pb sublattice. The atomic layers are labeled with the numbers. (b) The lattice parameter *c* map calculated from the ZrO/TiO sublattice. The atomic layers are labeled with the numbers. (c) The *z*-component map of displacement of the Pb columns respective to the neighboring ZrO/TiO columns. The atomic layers are labeled with the numbers. (d) The *z*-component displacement map of the ZrO/TiO columns respective to the Pb columns. The atomic layers are labeled with the numbers.

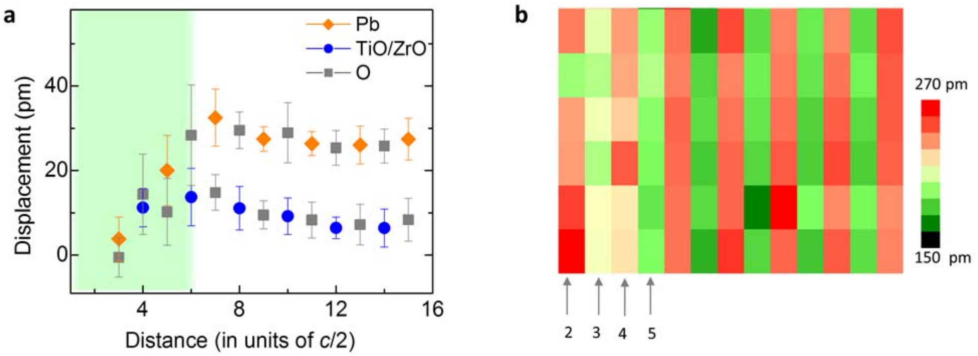

Supplementary Figure 2. | Structure of the negatively poled surface calculated from the cation and anion sublattices. (a) The average *z*-component of the displacement between the cation and anion columns including the displacement of the Pb columns respective to the neighboring O columns, the displacement of the ZrO/TiO columns respective to the neighboring O columns, and the displacement of the O columns respective to the neighboring Pb and ZrO/TiO columns. Six data points were averaged for Pb and one branch O displacements and five data points were averaged for ZrO/TiO and the other branch O displacements. The error bar is the standard deviation. The green color highlights the surface region with different atomic configuration. (b) The map of the Pb-O bond length along [001] direction. The numbers are the labels for atomic layers.

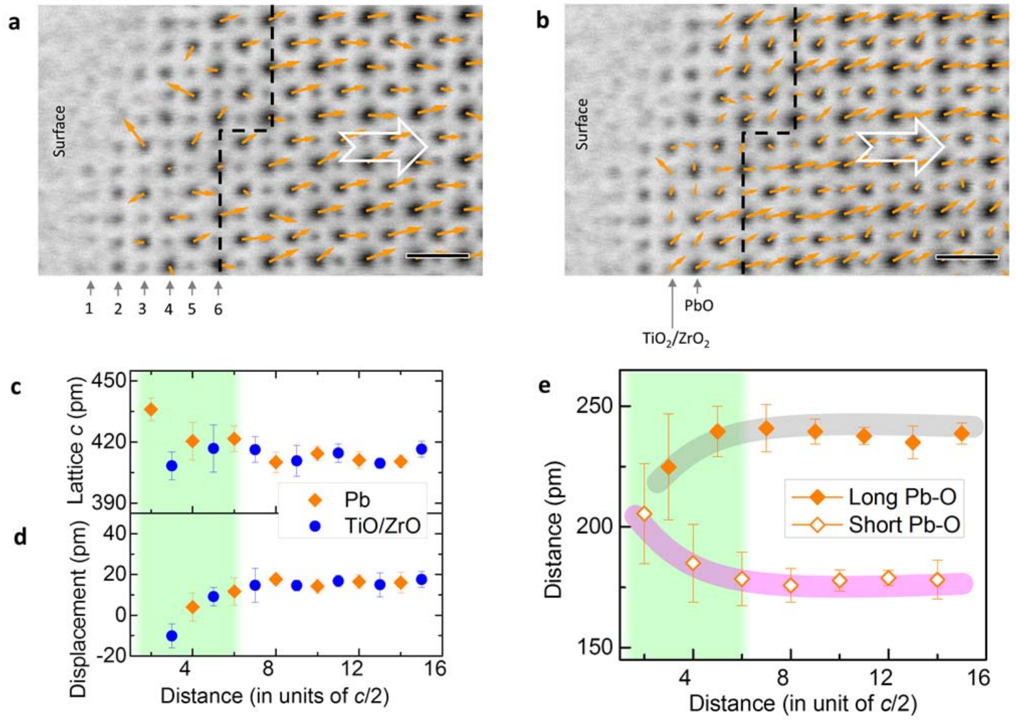

Supplementary Figure 3. | Structure of the negatively poled surface. (a) Displacement vectors between the Pb and ZrO/TiO columns overlaid with the ABF image. The atomic layers are labeled with the numbers. The arrow indicates the polarization is downward in this domain. The dashed line roughly highlights the boundary between the surface zone and bulk-like zone. Scale bar: 0.5 nm. (b) Vector map of the displacement between the cation and anion columns. The arrow indicates the polarization is downward in this domain. The dashed line roughly highlights the boundary between the surface zone and bulk-like zone. Scale bar: 0.5 nm. (c) Mean of the lattice parameter in [001] direction. Six data points were averaged to calculate the Pb lattice and five data points were averaged to plot ZrO/TiO lattice. The error bar is the standard deviation. The green color highlights the surface region with different atomic configuration. (d) The *z*-component of displacement. Six data points were averaged to plot Pb displacement and five data points were averaged to plot ZrO/TiO displacement. The error bar is the standard deviation. The green color highlights the surface region with different atomic configuration. (e) The long and short bond length of Pb-O along *z*-direction. Six data points were averaged to plot the Pb-O bond length. The error bar is the standard deviation. The green color highlights the surface region with different atomic configuration. The pink band highlights the shorter Pb-O length, which directly represents the magnitude of polarization in PZT. The grey band highlights the longer Pb-O length.

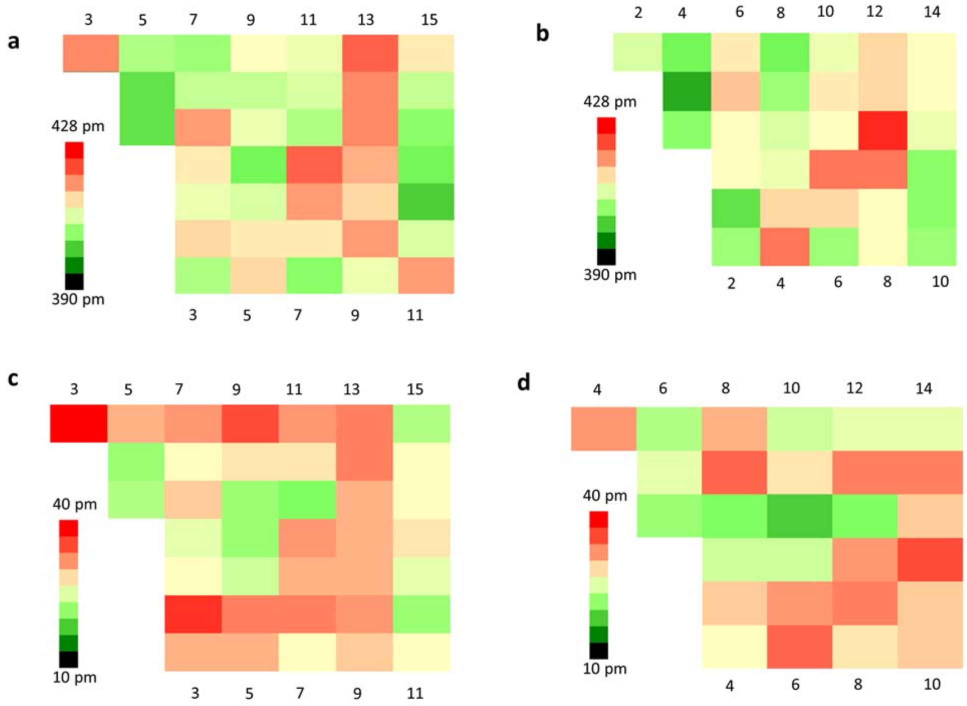

Supplementary Figure 4. | Structure of the positively poled surface calculated from the cation sublattice. (a) The lattice parameter *c* map calculated from the Pb sublattice. The atomic layers are labeled with the numbers. (b) The lattice parameter *c* map calculated from the ZrO/TiO sublattice. The atomic layers are labeled with the numbers. (c) The *z*-component map of the displacement of the Pb columns respective to the neighboring ZrO/TiO columns. The atomic layers are labeled with the numbers. (d) The *z*-component displacement map of the ZrO/TiO columns respective to the Pb columns. The atomic layers are labeled with the numbers.

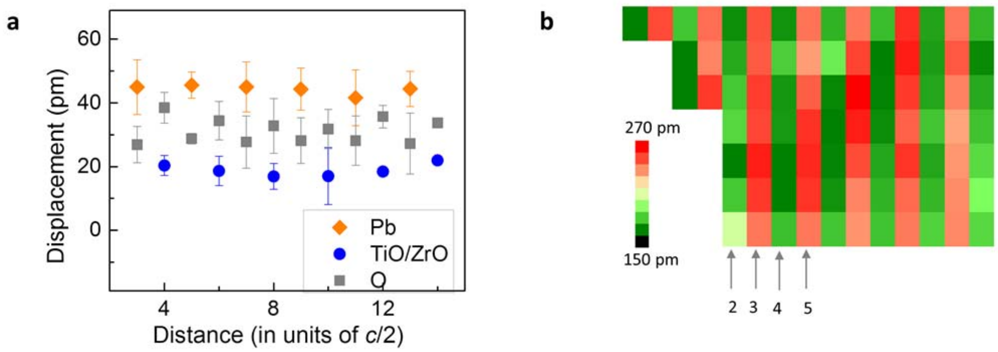

Supplementary Figure 5. | Structure of the positively poled surface calculated from the cation and anion sublattices. (a) The average *z*-component of the displacement between the cation and anion columns, including the displacement of the Pb columns respective to the neighboring O columns, the displacement of the ZrO/TiO columns respective to the neighboring O columns, and the displacement of the O columns respective to the neighboring Pb and ZrO/TiO columns. Seven data points were averaged for Pb and one branch O displacement and six data points were averaged for ZrO/TiO and the other branch O displacements. The error bar is the standard deviation. (b) Map of the Pb-O bond length along [001] direction. The atomic layers are labeled with the numbers.

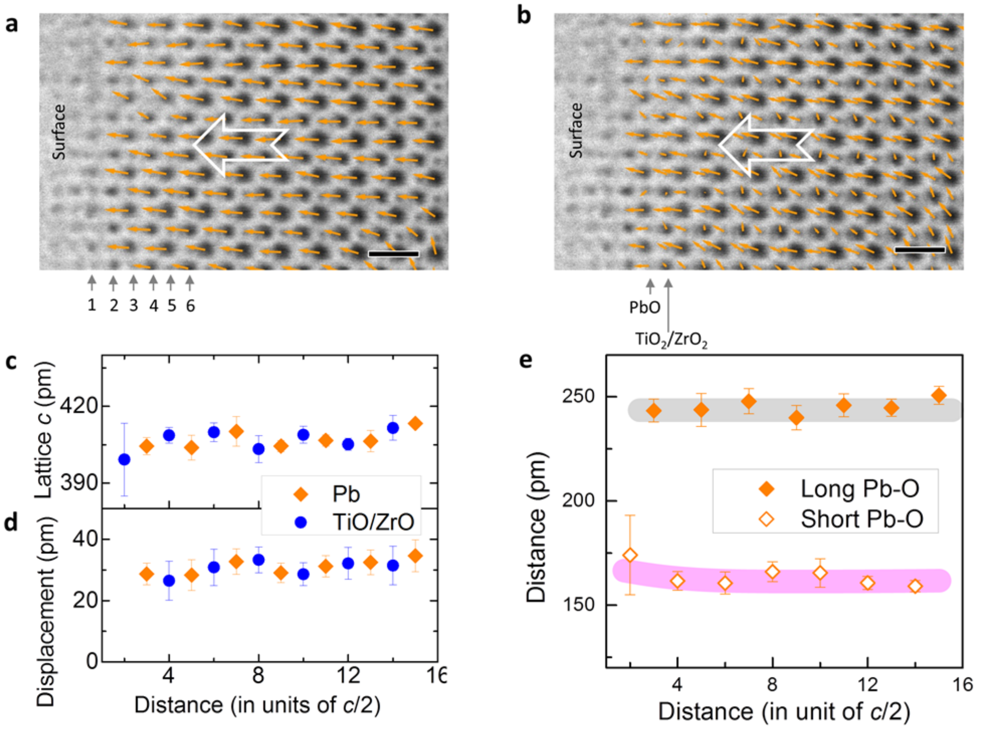

Supplementary Figure 6. | Structure of the positively poled surface. (a) The vector map of the displacement between the Pb and ZrO/TiO columns overlaid with the ABF image. The atomic layers are labeled with the numbers. The arrow indicates the polarization is upward in this domain. Scale bar: 0.5 nm. (b) The vector map of the displacement between the cation and anion columns. The arrow indicates the polarization is upward in this domain. Scale bar: 0.5 nm. (c) Mean of the lattice parameter *c* near the surface. Seven data points were averaged to plot Pb lattice and six data points were averaged to plot ZrO/TiO lattice. The error bar is the standard deviation. (d) Mean of the displacement between the Pb and ZrO/TiO columns. Seven data points were averaged to plot Pb displacement and six data points were averaged to plot ZrO/TiO displacement. The error bar is the standard deviation. (e) The long and short bond length of Pb-O along the *z*-direction. Seven data points were averaged to plot Pb-O bond length. The error bar is the standard deviation. The pink band highlights the shorter Pb-O length, which directly represents the magnitude of polarization in PZT. The grey band highlights the longer Pb-O length. No distinguishable difference is observed between the surface and subsurface.

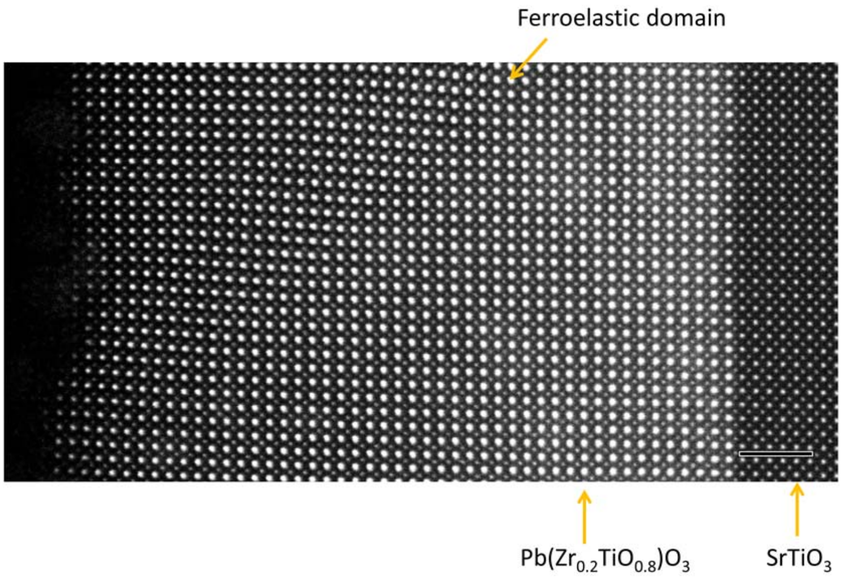

Supplementary Figure 7. | High angle annular dark field image of the $Pb(Zr_{0.2}Ti_{0.8})O_3$ thin film. The top arrow highlighting a ferroelastic domain, which is about 45° inclined to the substrate $SrTiO_3$. Scale bar: 2 nm.

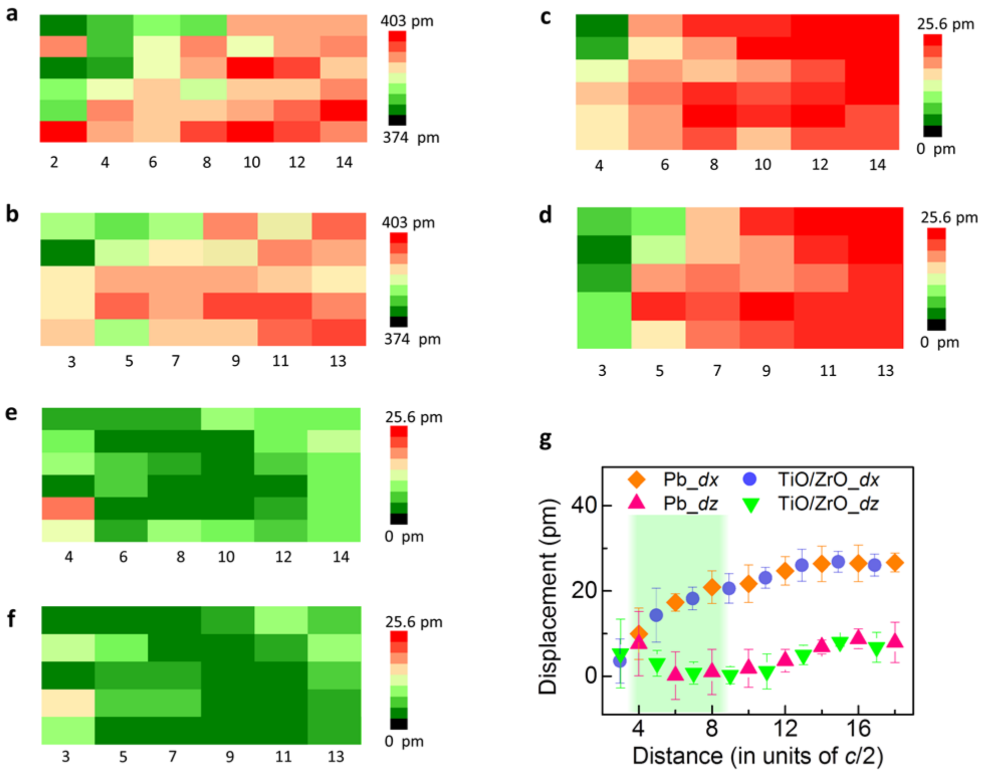

Supplementary Figure 8. | Structure of the ferroelastic domain calculated from the cation sublattice. (a) The lattice parameter *c* map calculated from the Pb sublattice. The atomic layers are labeled with the numbers. (b) The lattice parameter *c* map calculated from the ZrO/TiO sublattice. The atomic layers are labeled with the numbers. (c) The *x*-component displacement map of the Pb positions respective to the neighboring ZrO/TiO columns. The atomic layers are labeled with the numbers. (d) The *x*-component displacement map of the ZrO/TiO columns respectively to the neighboring Pb columns. The atomic layers are labeled with the numbers. (e) The *z*-component displacement map of the Pb positions respective to the neighboring ZrO/TiO columns. The atomic layers are labeled with the numbers. (f) The *z*-component displacement map of the ZrO/TiO columns respectively to the neighboring Pb columns. The atomic layers are labeled with the numbers. (g) The plot of displacement between the cation columns in two directions. Six data points were averaged for Pb_*dx* and Pb_*dz* displacements and five data points were averaged for ZrO/TiO_*dx* and ZrO/TiO_*dz* displacements. The error bar is the standard deviation. The green color highlights the surface region with different atomic configuration.

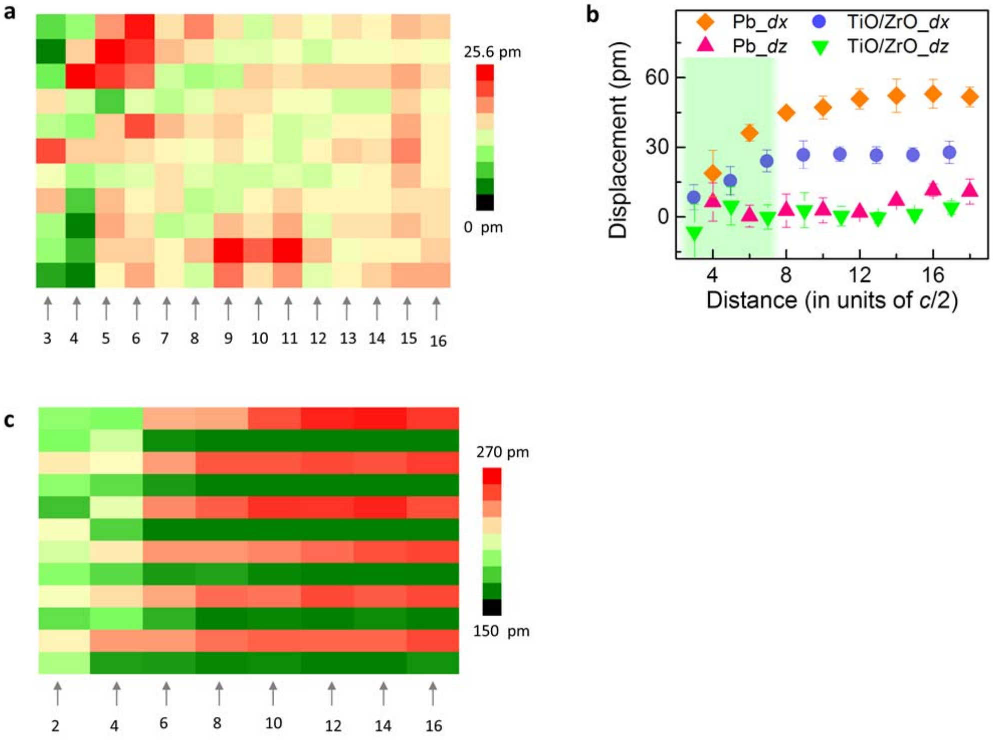

Supplementary Figure 9. | Structure of the ferroelastic domain surface calculated from the cation and anion sublattices. (a) The average *z*-component of the displacement between the cation and anion columns. The atomic layers are labeled with the numbers. (b) The plot of the displacement for two directions including the displacement of the Pb columns respective to the neighboring O columns, and the displacement of the ZrO/TiO columns respective to the neighboring O columns. Six data points were averaged for Pb_*dx* and Pb_*dz* displacement and five data points were averaged for ZrO/TiO_*dx* and ZrO/TiO_*dz* displacement. The error bar is the standard deviation. The green color highlights the surface region with different atomic configuration. (c) Map of the Pb-O bond length along [100] direction (in-plane polarization, *x*-component). The atomic layers are labeled with the numbers.

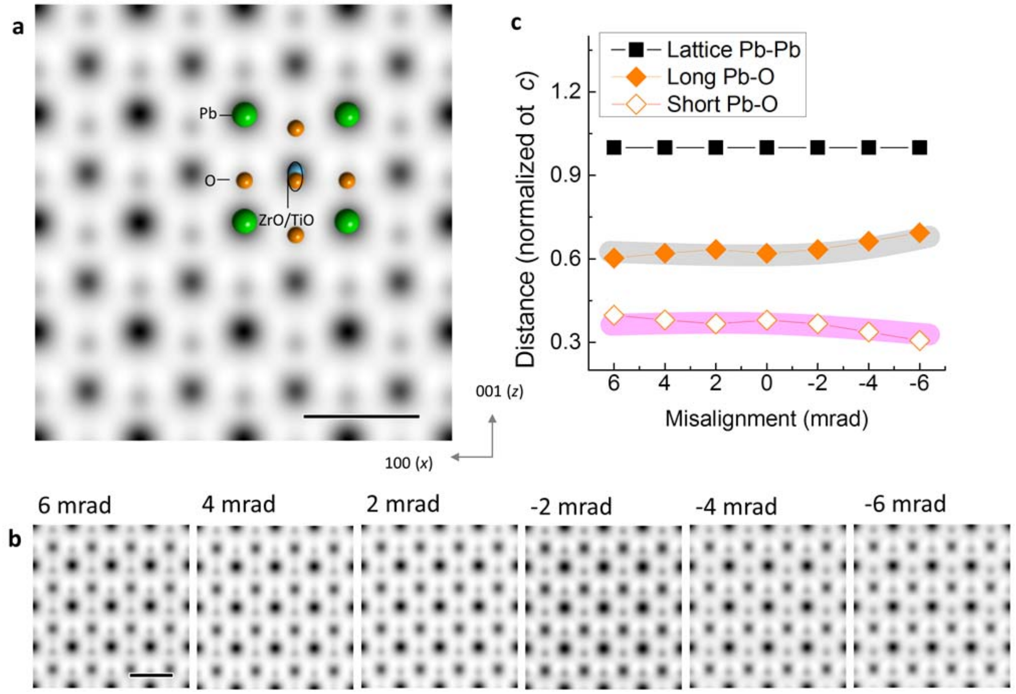

Supplementary Figure 10. | Image simulation of specimen tilt effect. (a) A simulated ABF image of $Pb(Zr_{0.2}Ti_{0.8})O_3$ structure. Scale bar: 0.5 nm. A schematic is overlaid on the simulated image. (b) With specimen tilt, simulated ABF images of $Pb(Zr_{0.2}Ti_{0.8})O_3$ structure. Scale bar: 0.5 nm. (c) The plots of lattice of Pb-Pb, long Pb-O, and short Pb-O bond length as a function of misalignment. The pink band highlights the shorter Pb-O length, and the grey band highlights the longer Pb-O length.

Supplementary Discussion.

1. Amorphous layer on the surface.

The specimens are inevitably coated by a thin amorphous layer after ion milling. To minimize the thickness of amorphous layer, our cross-sectional STEM specimens were cleaned by ion milling at 0.1 kV. The typical amorphous layer thickness in our samples is less than 3 nm. Owing to a very strong channeling effect on crystalline structure, we can visualize the atomic columns as dark dot contrast in ABF STEM images. While, the amorphous layer consists of random atom distribution and therefore the layers can contribute to simply increase the background noise level and not significantly change the atomic column positions. To determine the atomic position, we have implemented 2D Gaussian fitting, where we use more than 500 pixels per column. Therefore, the statistics is very high and the background noise attributed to the amorphous layer should be negligibly small.

2. Specimen tilt.

During experiments, we deliberately reduce the misalignment by means of:

i. The STEM mode was carefully aligned. All the data was recorded from the prototype JEM ARM300CF that has a very friendly and efficient alignment system. The aberration therefore can be corrected and minimized before recording data needless of standard specimen.

ii. We use CCD to align both specimen orientation and illumination aperture location on a big monitor by watching the Kikuchi patterns. We marked different angle contours such as 24 mrad, 6 mrad and 3 mrad on the monitor by home-made Digital Micrograph script.

iii. We used smaller illumination aperture (8 mrad) to doubly check the alignment. With the small aperture, a small misalignment can be more readily observed and corrected.

iv. We used larger illumination aperture (24 mrad) to record ABF images. Our multislice simulations and previous study [1] suggest that the effects of misalignment are less significant with large illumination aperture.

By these methods, we can easily make the misalignment as small as 3 mrad (equivalent to ~0.17°). In fact, in our experimental images in Figures 1a, 3a, and 4a, the atom columns appear "round" shape confirms that the alignment was very close to perfect zone axis. We note that our previous simulation [2] indicated ABF image is more sensitive to the misalignment compared to HAADF and small misalignment could significantly alter the shape of atomic columns.

Furthermore, we have checked the misalignment effects by multi-slice image simulation in Figure R1 that is shown below, and we are sure that the change of surface atom positions is an intrinsic behavior in this material rather than the artifacts from misalignment, which is discussed below.

i. From the simulation in Figure R1 the lattice constants that are calculated from interdistance of Pb-Pb columns remain unchanged regardless of the misalignment, which is not consistent with our results in Figures 2 and 4, confirming the presence of surface reconstruction instead of misalignment effects in our study. The constant interdistance of Pb-Pb columns can be interpreted by the fact that all the Pb columns show the same deformation behavior with specimen tilt and therefore the relative interdistance between them does not change at all regardless of misalignments.

ii. From the simulation in Figure R1, the misalignment indeed can influence on the length of short Pb-O bond, i.e., short Pb-O increases when tilt angle $\alpha>0$ and it decrease when $\alpha<0$. Note that only the former case $\alpha>0$ is possible in our experiments because short Pb-O bond length in the surface reconstructions never decreases in Figures 2, 3 and 4. Furthermore, within a misalignment of ±6 mrad, the increasement in short Pb-O bond length is less than 4.6% (at +6 mrad) from the simulation in Figure R1. Such subtle elongation (4.6%) is much smaller than that we observed in the Figures 2f and 4g, where the elongation of short Pb-O bond length is as large as 31%. In other words, the specimen misalignment caused increment in short Pb-O bond length (4.6%) can be even smaller than the error bar (as large as ±8.5%) in Figures 2f and 4g. In this sense, the effects of small specimen misalignment would not alter our conclusion.

Supplementary References

1. Y.-G. So, K. Kimoto, Effect of specimen misalignment on local structure analysis using annular dark-field imaging, Journal of Electron Microscopy **61**, 207-215 (2012).

2. S.D. Findlay, N. Shibata, H. Sawada, E. Okunishi, Y. Kondo, Y. Ikuhara, Dynamics of annular bright field imaging in scanning transmission electron microscopy, Ultramicroscopy **110**, 903-923 (2010).
